# [Retracted] Tetramethylpyrazine inhibits prostate cancer progression by downregulation of forkhead box M1

**DOI:** 10.3892/or.2024.8811

**Published:** 2024-09-16

**Authors:** Yi Zhou, Zhigang Ji, Weigang Yan, Zhien Zhou, Hanzhong Li, Yu Xiao

Oncol Rep 38: 837–842, 2017; DOI: 10.3892/or.2017.5768

Following the publication of this paper, it was drawn to the Editor's attention by a concerned reader that certain of the Transwell invasion assay data shown in Fig. 3B on p. 839 were strikingly similar to data that had already appeared in another article written by different authors at different research institutes [Mao Y, Zhang L, Yuan L, Yan M and He Y: MiR-218 suppresses cell progression by targeting APC in cervical cancer. Int J Clin Exp Pathol: 10, 2259-2269, 2017].

Owing to the fact that the contentious data in the above article had already been published prior to its submission to *Oncology Reports*, the Editor has decided that this paper should be retracted from the Journal. The authors were asked for an explanation to account for these concerns, but the Editorial Office did not receive a reply. The Editor apologizes to the readership for any inconvenience caused.

